# Phenotypic characterization of ENPP1 deficiency: generalized arterial calcification of infancy and autosomal recessive hypophosphatemic rickets type 2

**DOI:** 10.1093/jbmrpl/ziaf019

**Published:** 2025-01-30

**Authors:** Carlos R Ferreira, Mary E Hackbarth, Yvonne Nitschke, Ulrike Botschen, Rachel I Gafni, M Zulf Mughal, Genevieve Baujat, Dirk Schnabel, I Manjula Schou, Gus Khursigara, Oona Reardon, Thomas R Burklow, Kathleen Swanner, Frank Rutsch

**Affiliations:** Eunice Kennedy Shriver National Institute of Child Health and Human Development, National Institutes of Health, Bethesda, MD 20892, United States; National Human Genome Research Institute, National Institutes of Health, Bethesda, MD 20892, United States; Department of General Pediatrics, Münster University Children’s Hospital, 48149 Münster, Germany; Department of General Pediatrics, Münster University Children’s Hospital, 48149 Münster, Germany; National Institute of Dental and Craniofacial Research, National Institutes of Health, Bethesda, MD 20892, United States; Al Jalila Children’s Specialty Hospital, Dubai, UAE; Département de Génétique, Centre de Référence Maladies Osseuses Constitutionnelles (CR MOC) et Filière OSCAR, Hôpital Necker-Enfants Malades, 75015 Paris, France; Center for Chronically Sick Children, Pediatric Endocrinology, Charitè, University Medicine Berlin, 10117 Berlin, Germany; Pulse Economics Pty Ltd., Macquarie Park, NSW 2113, Australia; Inozyme Pharma, Inc., Boston, MA 02210, United States; Pulse Economics Pty Ltd., Macquarie Park, NSW 2113, Australia; NIH Clinical Center, National Institutes of Health, Bethesda, MD 20892, United States; Inozyme Pharma, Inc., Boston, MA 02210, United States; Department of General Pediatrics, Münster University Children’s Hospital, 48149 Münster, Germany

**Keywords:** disorders of calcium/phosphate metabolism, diseases and disorders of/related to bone, osteomalacia and rickets, epidemiology, genetics

## Abstract

Generalized arterial calcification of infancy (GACI) and autosomal recessive hypophosphatemic rickets type 2 (ARHR2) are age-related phenotypes of the rare genetic mineralization disorder, ENPP1 Deficiency, which evolve on a phenotypic continuum. To date, our understanding of the clinical spectrum of ENPP1 Deficiency is based on small studies or case reports, across which there is significant variability in clinical presentation, and limited duration of follow-up. From a previously published large retrospective natural history study, we performed a subgroup analysis to elucidate the most prevalent signs and symptoms of ENPP1 Deficiency diagnosed as GACI or ARHR2, to illustrate the onset and incidence of these complications over the lifetime, and to characterize the associated medical burden of disease. Of the 84 individuals with ENPP1 Deficiency analyzed, 51 had a recorded diagnosis of GACI, 11 were diagnosed with ARHR2, and 22 were diagnosed with both. We confirmed that those diagnosed with GACI presented predominantly with early-onset arterial calcification, respiratory distress, heart failure, and hypertension, necessitating acute inpatient care and leading to high (44%) infant mortality. Notably, we found that the majority (60.3%) of those with a history of GACI had prenatal ultrasound anomalies, including effusions, polyhydramnios, and hydrops fetalis. We estimated that 70% of individuals with ENPP1 Deficiency who survive to age 10 will have developed musculoskeletal complications, primarily rickets and/or osteomalacia. The clinical picture of ARHR2 in this study extended beyond skeletal deformities to include hearing impairment, joint involvement, and ongoing risk of cardiovascular problems. This study sheds light on the signs and symptoms of ENPP1 Deficiency in the real world, with implications for life-long patient monitoring.

## Graphical Abstract

**Graphical Abstract:**
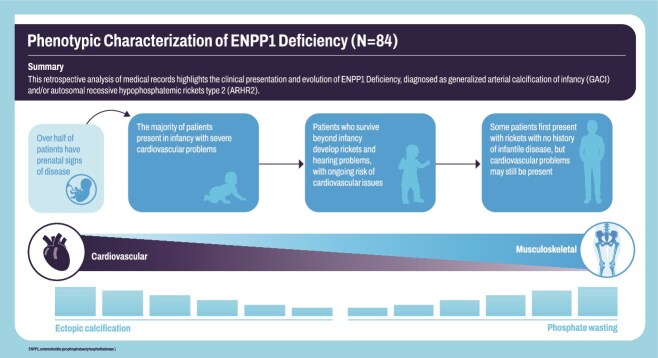

## Introduction

Ectonucleotide pyrophosphatase/phosphodiesterase family member 1 (ENPP1) is a transmembrane glycoprotein that catalyzes the hydrolysis of extracellular adenosine triphosphate (ATP), generating inorganic pyrophosphate (PP_i_) and adenosine monophosphate (AMP).^1^ Both of these metabolites play important homeostatic functions in bone health and blood vessel function. Inorganic pyrophosphate is a key regulator of bone and soft tissue mineralization that acts by inhibiting the formation and propagation of hydroxyapatite crystals.^2^ Adenosine monophosphate, the other by-product of ATP metabolism by ENPP1, is further converted to adenosine and phosphate (P_i_) by CD73.^3^ Both AMP and adenosine are important regulators of vascular smooth muscle cell proliferation.^3^

ENPP1 Deficiency is a rare condition linked to disruption of the PP_i_-adenosine pathway.^4^ It is caused by biallelic pathogenic variants in the *ENPP1* gene, leading to loss of enzymatic activity and consequently low levels of plasma PP_i_ and adenosine, ectopic calcification, skeletal under-mineralization, and occlusive arterial neointimal proliferation.^5^ There are 2 primary phenotypes of biallelic ENPP1 Deficiency, largely associated with age of onset:

Generalized arterial calcification of infancy (GACI) type 1, which typically manifests in early infancy, is characterized by arterial calcification that is life-threatening in approximately 50% of infants^6^; and
Autosomal recessive hypophosphatemic rickets type 2 (ARHR2), which presents during childhood and is characterized by FGF23-mediated hypophosphatemia.^7^

Importantly, available evidence suggests that the majority of GACI survivors develop FGF23-mediated hypophosphatemia in childhood, thus making GACI and ARHR2 a phenotypic continuum or single spectrum of disease.^8^ Despite this, GACI and ARHR2 populations are often described separately in the literature, with a focus on the clinical presentation prompting diagnosis, and in the absence of long-term follow-up.^9^ Beyond arterial calcification and rickets/osteomalacia, previous studies demonstrate that GACI and ARHR2 phenotypes are associated with widespread systemic clinical complications.^10^ There is considerable variability in clinical presentation between individual patients, even among siblings with the same variants.^5^^,^^7^ While previous publications have examined possible associations, no genotype-phenotype correlations have been reported to date.^9^ Detailed characterization of the manifestations and phenotypic evolution of ENPP1 Deficiency serve to inform diagnosis and clinical management.

The primary objective of this retrospective natural history study in a large population with ENPP1 Deficiency was to characterize the onset and cumulative incidence of the clinical complications of ENPP1 Deficiency over time, grouped into 4 core categories: calcification, cardiovascular, musculoskeletal, and other organ involvement.^10^ Additionally, we describe the clinical manifestations and medical burden of ENPP1 Deficiency diagnosed as GACI-only, GACI and ARHR2, or ARHR2-only.

## Materials and methods

### Study design and individuals

This was a retrospective subgroup analysis of a previously described large natural history study compiled from 2 primary chart review studies (NCT03478839 and NCT03758534).^10^ Study details, including ethics approval and consent, have been described previously.^10^ This analysis included individuals with a diagnosis of GACI and/or ARHR2, confirmed by imaging, biochemical findings, and/or *ENPP1* sequencing. Individuals were classified as GACI-only if they were not affected with ARHR2 at the time of data collection, or their ARHR2 status was unknown. Individuals were classified as GACI and ARHR2 if both diagnoses were reported in the case report forms (CRFs). Finally, individuals were classified as ARHR2-only if a diagnosis of ARHR2 and no diagnosis of GACI was reported. For each individual, relevant information was collected from before birth to the date of death, or loss to follow-up, whichever occurred first. The analysis included data from individuals born between 1960 and 2018 (median 2010) and retrieved between 2018 and 2020.

### Procedures

Case report forms were developed for the medical chart review. The CRFs included details on vital status, diagnosis, mutational analysis, past birth/medical histories, initial postnatal presentation with GACI, clinical course, therapies, and laboratory results. Where only month and year of birth were recorded in the CRF, date of birth was imputed to be the first of the month.

### Statistical analysis

Descriptive statistics including means, SD, medians, minimums and maximums for continuous variables, and numbers and percentages for categorical variables were used to summarize patient and disease characteristics. Where time to event was measured for reporting, descriptive statistics needed to consider both the duration of time to event in those who experienced the event, and the duration of follow-up in those who did not experience the event. Therefore, these data were summarized using cumulative incidence analyses, which account for both the time and event status (ie, event did or did not occur by the time of last follow-up). These were generated for ectopic calcifications and cardiovascular, musculoskeletal, and other organ manifestations. Where a date was not attributed to a particular event, the patient’s age at last follow-up was used. Individuals who did not experience the event of interest contributed a censored observation, including those who died without experiencing the event. The number “at risk” reduces over time as the time-to-event or follow-up end, whichever is the earlier, occurs. Therefore, the estimates of these time-to-event summaries are less robust where the numbers at risk are low.

## Results

### Overall population—demographics

Eighty-four individuals were included in the analysis, with baseline demographics reflecting a generally young ENPP1 Deficiency cohort presenting with severe, early-onset disease. As shown in Table 1, the median age was 13 d at symptom onset and 42 d at diagnosis. In those who were alive at the time of last follow-up, the median age was approximately 9 yr (range 4-18 yr); overall, 40.5% had died over the follow-up period. The majority (73/84; 86.9%) had a recorded diagnosis of GACI, of whom 33 died (39.3%). In those diagnosed with GACI who were alive at last follow-up, 21/40 (52.5%) were subsequently diagnosed with ARHR2 (GACI and ARHR2). In addition, 11 were first diagnosed with ARHR2 with no recorded GACI diagnosis. Overall, 17.9% of individuals had living siblings with a diagnosis of GACI and 22.6% had deceased siblings with a diagnosis of GACI (Table S1). The clinical complications recorded in individuals diagnosed with GACI-only, ARHR2-only, or both phenotypes are captured in detail in the subsequent sections.

**Table 1:** Participant demographics.

**Characteristic**	**Overall (*N* = 84)**	**GACI-only (*n* = 51)**	**GACI and ARHR2 (*n* = 22)**	**ARHR2-only (*n* = 11)**
**Gestational age, wk** ***N*** ** Median (Q1, Q3)**	75 36.0 (33.2, 37.1)	49 35.3 (33.0, 36.7)	20 36.3 (33.8, 38.0)	6 38.0 (36.3, 39.8)
**Age at symptom onset, mo** *** N*** ** Median (Q1, Q3)**	65 0.4 (0.0, 0.8)	40 0.3 (0.0, 0.6)	19 0.2 (0.0, 0.8)	6 43.1 (18.4, 58.3)
**Age at diagnosis,** ^**a**^ **mo** *** N*** ** Median (Q1, Q3)**	81 1.4 (0.2, 31.6)	48 0.8 (0.1, 2.9)	22 1.0 (0.0, 25.8)	11 137.7 (54.3, 296.8)
**Sex, *n* (%)** ** Female**	41 (48.8)	21 (41.2)	15 (68.2)	5 (45.5)
**Survival status, *n* (%)** ** Alive** ** Deceased**	50 (59.5) 34 (40.5)	19 (37.3) 32 (62.7)	21 (95.5) 1 (4.5)	10 (90.9) 1 (9.1)
**Birth status, *n* (%)** ** In utero death** ** Live birth** ** Stillborn**	2 (2.4) 81 (96.4) 1 (1.2)	2 (3.9) 48 (94.1) 1 (2.0)	0 (0.0) 22 (100.0) 0 (0.0)	0 (0.0) 11 (100.0) 0 (0.0)
**Age at death, mo** ^**b**^ *** N*** ** Median (Q1, Q3)**	29 1.9 (0.7, 3.3)	27 1.8 (0.7, 2.7)	1 61.8 (61.8, 61.8)	1 58.1 (58.1, 58.1)
**Age at last follow-up, mo (in those alive)** *** N*** ** Median (Q1, Q3)**	50 111.1 (47.4, 214.2)	19 60.7 (28.1, 174.9)	21 117.6 (62.0, 228.4)	10 219.4 (147.3, 323.7)

a Missing date of diagnosis imputed with date of death (in deceased individuals) or last date of follow-up.

b Nonmissing dates of birth and death.

Abbreviations: ARHR2, autosomal recessive hypophosphatemic rickets type 2; GACI, generalized arterial calcification of infancy; Q, quartile.

### Overall population—time to detection of event analysis

**Figure 1:**
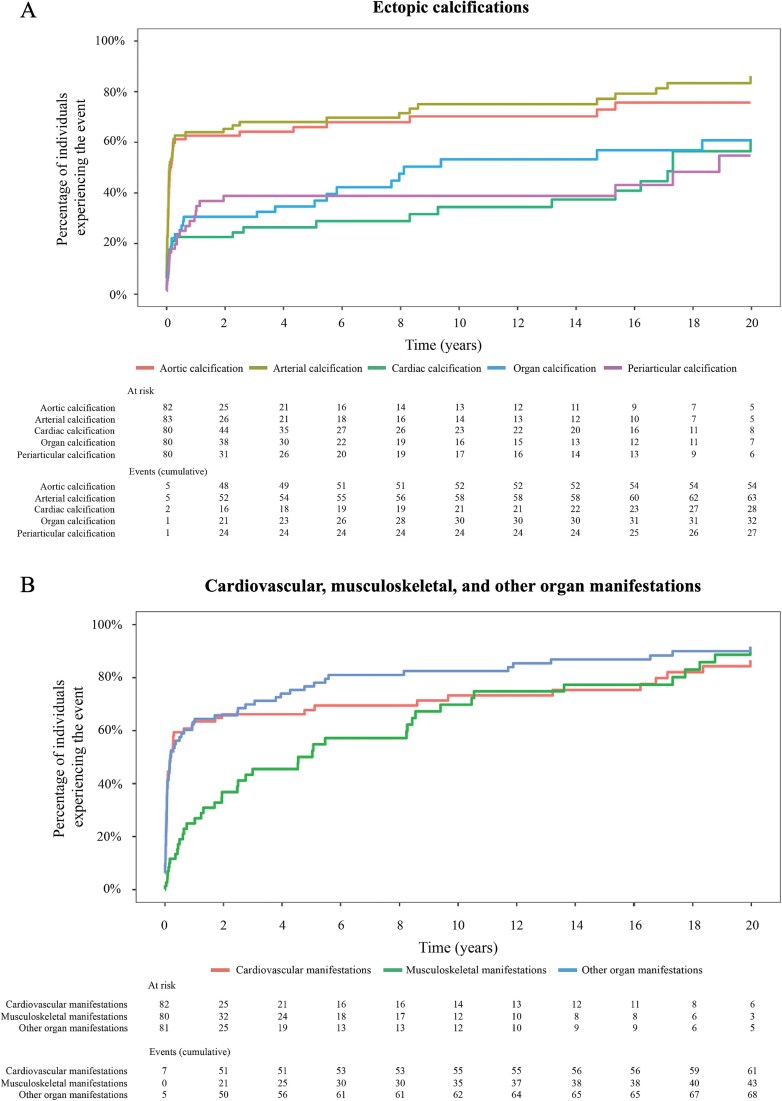
(A) Cumulative incidence of ectopic calcification over the first 20 yr of life; (B) cumulative incidence of cardiovascular, musculoskeletal, and other organ manifestations over the first 20 yr of life.

Across the overall population, a high incidence of ectopic calcifications and cardiovascular, musculoskeletal, and other organ manifestations were observed (Table 2A-F). Time-to-event analyses over the first 20 yr of follow-up were undertaken to characterize the onset and incidence of these manifestations in individuals at risk (Figure 1, Figures S1 and S2).

**Table 2:** Clinical manifestations.

**Manifestation, *n* (%)**	**Overall (*N* = 84)**	**GACI-only (*n* = 51)**	**GACI and ARHR2 (*n* = 22)**	**ARHR2-only (*n* = 11)**
**A. Prenatal findings**				
**Any**	44 (52.4)	31 (60.8)	13 (59.1)	0 (0.0)
**Effusions**	20 (23.8)	14 (27.5)	6 (27.3)	0 (0.0)
**Polyhydramnios**	20 (23.8)	16 (31.4)	4 (18.2)	0 (0.0)
**Presence of calcifications of arteries**	18 (21.4)	9 (17.6)	9 (40.9)	0 (0.0)
**Hydrops fetalis**	13 (15.5)	10 (19.6)	3 (13.6)	0 (0.0)
**Presence of calcifications of organs**	7 (8.3)	5 (9.8)	2 (9.1)	0 (0.0)
**Cardiomegaly**	5 (6.0)	5 (9.8)	0 (0.0)	0 (0.0)
**Cardiac failure**	3 (3.6)	2 (3.9)	1 (4.5)	0 (0.0)
**Stenosis of arteries**	2 (2.4)	0 (0.0)	2 (9.1)	0 (0.0)
**Presence of calcifications of joints**	1 (1.2)	1 (2.0)	0 (0.0)	0 (0.0)
**B. Postnatal arterial stenosis**				
**Stenosis of arteries**	9 (10.7)	8 (15.7)	1 (4.5)	0 (0.0)
**C. Postnatal calcification**				
**Any**	74 (88.1)	45 (88.2)	22 (100.0)	7 (63.6)
**Arterial calcification**^**a**^	68 (81.0)	43 (84.3)	22 (100.0)	3 (27.3)
**Aortic calcification**^**b**^	56 (66.7)	36 (70.6)	19 (86.4)	1 (9.1)
**Organ calcification**^**c**^	34 (40.5)	22 (43.1)	11 (50.0)	1 (9.1)
**Periarticular calcification**^**d**^	31 (36.9)	15 (29.4)	13 (59.1)	3 (27.3)
**Cardiac calcification**^**e**^	30 (35.7)	16 (31.4)	11 (50.0)	3 (27.3)
**Calcified entheses**^**r**^	3 (3.6)	0 (0.0)	2 (9.1)	1 (9.1)
**D. Postnatal cardiovascular manifestations**				
**Any**	64 (76.2)	41 (80.4)	16 (72.7)	7 (63.6)
**Hypertension**	43 (51.2)	29 (56.9)	10 (45.5)	4 (36.4)
**Heart valve defect**	26 (31.0)	17 (33.3)	5 (22.7)	4 (36.4)
**Signs of left ventricular hypertrophy**	25 (29.8)	22 (43.1)	3 (13.6)	0 (0.0)
**Cardiac failure**	23 (27.4)	20 (39.2)	2 (9.1)	1 (9.1)
**Pericardial effusion**	17 (20.2)	13 (25.5)	4 (18.2)	0 (0.0)
**Cardiomegaly**^**f**^	15 (17.9)	12 (23.5)	2 (9.1)	1 (9.1)
**Reduced ejection fraction**	13 (15.5)	11 (21.6)	2 (9.1)	0 (0.0)
**Cyanosis**	13 (15.5)	9 (17.6)	3 (13.6)	1 (9.1)
**Cardiomyopathy**^**g**^	10 (11.9)	7 (13.7)	3 (13.6)	0 (0.0)
**Pulmonary hypertension**	10 (11.9)	8 (15.7)	2 (9.1)	0 (0.0)
**E. Postnatal musculoskeletal manifestations**				
**Any**	46 (54.8)	13 (25.5)	22 (100.0)	11 (100.0)
**Rickets**	36 (42.9)	4 (7.8)	21 (95.5)	11 (100.0)
**Bowed extremities**	26 (31.0)	3 (5.9)	15 (68.2)	8 (72.7)
**Pain**	23 (27.4)	2 (3.9)	15 (68.2)	6 (54.5)
**Metaphyseal abnormalities**^**h**^	23 (27.4)	6 (11.8)	13 (59.1)	4 (36.4)
**Joint pain**	17 (20.2)	8 (15.7)	6 (27.3)	3 (27.3)
**Gait abnormality**^**i**^	14 (16.7)	4 (7.8)	7 (31.8)	3 (27.3)
**Short stature**	12 (14.3)	1 (2.0)	8 (36.4)	3 (27.3)
**Fracture**	5 (6.0)	2 (3.9)	2 (9.1)	1 (9.1)
**Osteopenia**^**r**^	5 (6.0)	1 (2.0)	2 (9.1)	2 (18.2)
**Osteolytic changes**	4 (4.8)	0 (0.0)	3 (13.6)	1 (9.1)
**Vertebral fusion**^**r**^	4 (4.8)	1 (2.0)	2 (9.1)	1 (9.1)
**F. Postnatal other organ manifestations**				
**Any**	72 (85.7)	42 (82.4)	21 (95.5)	9 (81.8)
**Gastrointestinal**^**j**^	44 (52.4)	25 (49.0)	14 (63.6)	5 (45.5)
**Pulmonary dysfunction with/without edema**	41 (48.8)	31 (60.8)	9 (40.9)	1 (9.1)
**Renal**^**k**^	32 (38.1)	21 (41.2)	8 (36.4)	3 (27.3)
**Hearing**^**l**^	31 (36.9)	12 (23.5)	15 (68.2)	4 (36.4)
**Neurological**^**m**^	28 (33.3)	18 (35.3)	8 (36.4)	2 (18.2)
**Ophthalmologic**^**n**^	15 (17.9)	7 (13.7)	6 (27.3)	2 (18.2)
**Dermatological**^**o**^	12 (14.3)	7 (13.7)	4 (18.2)	1 (9.1)
**Pneumonia**	10 (11.9)	4 (7.8)	5 (22.7)	1 (9.1)
**G. Postnatal health care**				
**Hospitalization at presentation, *n* (%)**				
**Yes**	64 (76.2)	45 (88.2)	19 (86.4)	0 (0.0)
**No**	11 (13.1)	3 (5.9)	1 (4.5)	7 (63.6)
**Unknown**	9 (10.7)	3 (5.9)	2 (9.1)	4 (36.4)
**Age at hospitalization, d**				
***N***	57	41	16	0
**Median (Q1, Q3)**	10.0 (0.0, 22.0)	10.00 (0.0, 22.0)	10.5 (4.0, 24.5)	N/A (N/A, N/A)
**Duration of hospitalization, d**^**s**^				
***N***	52	38	14	0
**Median (Q1, Q3)**	28.0 (11.0, 44.3)	29.5 (11.0, 43.0)	24.0 (12.0, 42.0)	N/A (N/A, N/A)
**Manifestations at initial hospitalization, *n* (%)**				
**Dyspnea**^**p**^	49 (58.3)	40 (78.4)	9 (40.9)	0 (0.0)
**Cardiac insufficiency**	22 (26.2)	18 (35.3)	4 (18.2)	0 (0.0)
**Cyanosis**	18 (21.4)	15 (29.4)	3 (13.6)	0 (0.0)
**Pleural effusions**	7 (8.3)	5 (9.8)	2 (9.1)	0 (0.0)
**High blood pressure**	6 (7.1)	6 (11.8)	0 (0.0)	0 (0.0)
**Pulmonary edema**	4 (4.8)	3 (5.9)	1 (4.5)	0 (0.0)
**Jaundice**	4 (4.8)	4 (7.8)	0 (0.0)	0 (0.0)
**Seizures**	3 (3.6)	3 (5.9)	0 (0.0)	0 (0.0)
**Myocardial infarction**	2 (2.4)	1 (2.0)	1 (4.5)	0 (0.0)
**Bleeding**	2 (2.4)	1 (2.0)	1 (4.5)	0 (0.0)
**Syncope**	1 (1.2)	1 (2.0)	0 (0.0)	0 (0.0)
**Other**^**q**^	64 (76.2)	40 (78.4)	20 (90.9)	4 (36.4)
**Medical assessments, *n* (%)**				
**Echocardiogram**	60 (71.4)	39 (76.5)	17 (77.3)	4 (36.4)
**Ultrasound (postnatal)**	59 (70.2)	38 (74.5)	17 (77.3)	4 (36.4)
**Radiograph**	57 (67.9)	37 (72.5)	15 (68.2)	5 (45.5)
**CT**	35 (41.7)	21 (41.2)	13 (59.1)	1 (9.1)
**Initial postnatal GACI test added**	22 (26.2)	16 (31.4)	6 (27.3)	0 (0.0)
**Ophthalmologic examination**	21 (25.0)	11 (21.6)	9 (40.9)	1 (9.1)
**Hearing test**	21 (25.0)	10 (19.6)	10 (45.5)	1 (9.1)
**MRI**	15 (17.9)	14 (27.5)	1 (4.5)	0 (0.0)
**CT angiography**	9 (10.7)	9 (17.6)	0 (0.0)	0 (0.0)
**Arterial biopsy**	5 (6.0)	5 (9.8)	0 (0.0)	0 (0.0)

a Includes coronary, radial, iliac, renal, ulnar, femoral, popliteal, cerebral, mesenteric, splenic, axillary, brachial, bronchial, carotid, innominate, subclavian, pulmonary arteries, and other arterial calcification.

b Includes aorta, ascending aorta, aortic arch, descending aorta, and abdominal aorta.

c Includes lungs, liver, kidneys, intestines, and other organ calcification.

d Includes joint calcifications: shoulder, elbow, wrist, fingers, carpal, hip, knee, ankle, toes, mandible, sternoclavicular, and other joint calcification.

e Includes the myocardium and heart valve.

f Not collected in the data set as a separate variable. Instead, it was identified through a review of free-text fields capturing details (postnatal) of the patient’s disease characteristics collected in the data set.

g Not collected as a separate variable, identified through the term “cardiomyopathy” entered into the case report form.

h Includes metaphyseal cupping, metaphyseal irregularities, and other metaphyseal changes.

i Includes skeletal gait abnormality and gait abnormality.

j Includes nausea and vomiting, dyspepsia and/or indigestion, diarrhea, bloating and constipation, gastrointestinal bleeding, jaundice, feeding intolerance, and any other gastrointestinal findings.

k Includes renal dysfunction, proteinuria, aminoaciduria, albuminuria, polyuria, oligouria, increased creatinine, renal dialysis, and other renal dysfunction.

l Includes conductive hearing loss, sensorineural hearing loss, ankylosis of auditory ossicles, calcification of the ear cartilage, and any other hearing signs.

m Includes stroke, seizure, epilepsy, developmental delay, paralysis, and any other neurological signs.

n Includes angioid streaks, macular edema, retinal hemorrhage, choroidal neovascularization, and any other ophthalmologic signs.

o Includes pseudoxanthoma, erythema, papules, dermatologic plaques, Peau D’Orange, and any other dermatological signs.

p Includes respiratory distress.

q Includes other important manifestations at presentation.

r Identified by reviewing free-text fields.

s Includes the day of discharge.

Abbreviations: ARHR2, autosomal recessive hypophosphatemic rickets type 2; GACI, generalized arterial calcification of infancy; Q, quartile.

Most individuals (70%, 95% CI: 53%-81%) who survived beyond infancy were predicted to develop musculoskeletal complications (primarily rickets) by 10 yr of age (Figure 1, Table 2E). Arterial calcification and cardiovascular complications were reported in over 76% of the overall population, with an onset predominately during infancy (Table 2C and D, Figure 1). At least 60% of at-risk individuals had developed ectopic arterial calcification (61%, 95% CI: 49%-71%) or aortic calcification (60%, 95% CI: 47%-69%) by 3 mo of age (Figure S1). Vascular calcifications were first reported beyond infancy in approximately 28% of individuals across their lifetime. By 10 yr of age, 70% had ectopic arterial calcification (95% CI: 57%-79%) and 75% had aortic calcification (95% CI: 63%-83%).

Periarticular, cardiac, or organ calcifications were less prevalent than vascular calcifications in the first year of life but continued to be identified beyond infancy. The cumulative incidence of periarticular calcification rose from 31% (95% CI: 18%-42%) at 1 yr to 39% (95% CI: 25%-50%) by age 10 yr. The cumulative incidence of cardiac and other organ calcifications followed a similar trend, rising from 23% in cardiac (95% CI: 12%-32%) and 31% in other organs (95% CI: 19%-41%) at 1 yr to 34% (95% CI: 20%-46%) and 53% (95% CI: 36%-66%), by 10 yr.

Forty-six individuals (54.8%) had a medical history of musculoskeletal manifestations, predominately rickets, as outlined in Table 2E. A cumulative incidence analysis, censoring individuals who had died or had not experienced the event during the follow-up, indicated that the majority (52%) had developed rickets before age 10 yr. Musculoskeletal complications outlined in Table 2E were first observed in childhood (from infancy to adolescence). Surprisingly, musculoskeletal manifestations were recorded in some individuals as early as birth, with a cumulative incidence of 25% (95% CI: 13%-35%) by 1 yr. The cumulative incidence of this complication rose beyond infancy to 46% (95% CI: 30%-58%) by age 3 yr, 57% (95% CI: 41%-69%) by age 6, and 75% (95% CI: 58%-85%) by age 11 yr.

By age 55, we estimated that over 95% of surviving patients with ENPP1 Deficiency will have developed cardiovascular, musculoskeletal, and/or other organ complications (Figures S3 and S4).

### Clinical manifestations by phenotype

#### GACI-only

The GACI-only diagnosis cohort (*n* = 51) was comprised of clinically affected infants who succumbed to early mortality (62.7%) or who were alive at last follow-up and had not yet developed hypophosphatemic rickets during the observational period (median age of 5 yr in these survivors; Table 1). There were 32 individuals in this cohort who died at a median age of 1.8 mo, including 2 intrauterine fetal deaths and 1 stillbirth.

Abnormal prenatal ultrasound findings were evident in most (60.8%) individuals, identified primarily during the third trimester, but seen as early as 20 wk’ gestation in some. As depicted in Table 2A, the most common prenatal findings were polyhydramnios (31.4%), effusions (27.5%), and hydrops fetalis (19.6%), followed by arterial calcification (17.6%). Of the 31 patients with recorded prenatal ultrasound anomalies, only 2 were diagnosed with GACI prenatally, 1 with evidence of calcification.

The median gestational age at birth in individuals with a GACI-only diagnosis was 35.3 wk, and 37 (72.5%) were born prematurely (<37 wk’ gestation): 25 via C-section, 8 spontaneous deliveries, 1 induced delivery, 1 forceps delivery, and 2 unknown.

**Figure 2:**
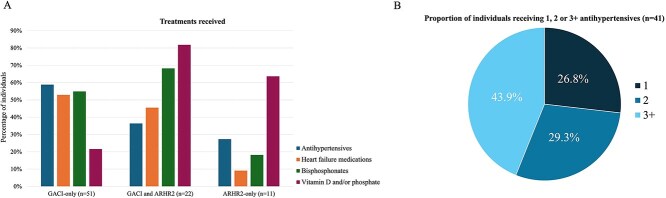
(A) Pharmacological treatments received by diagnosis; (B) proportion of the total population treated for hypertension who received 1, 2, or 3 or more antihypertensive medications, *n* = 41. Antihypertensive medications included angiotensin-converting enzyme (ACE) inhibitors, beta blockers, calcium channel blockers, thiazide diuretics, vasodilators, adrenergic receptor blockers, angiotensin receptor blockers, renin inhibitors, and mineralocorticoid receptor antagonist. Heart failure medications included diuretics, ACE inhibitors, beta blockers, aspirin, calcium channel blockers, digoxin, hydralazine, mineralocorticoid receptor antagonist, and nitrate.

The median age at first recorded sign or symptom was 9.5 d (range: 0-19 d; Table 1). The majority (88.2%) of individuals were hospitalized at initial presentation, primarily with respiratory distress (dyspnea [78.4%] and cyanosis [29.4%]), and 80.4% required mechanical ventilation (Table 2G). The most prevalent postnatal signs and symptoms recorded at any time point were ectopic arterial (84.3%) and aortic (70.6%) calcification with heterogenous cardiovascular manifestations (80.4%), including hypertension (56.9%), left ventricular hypertrophy (43.1%), cardiac failure (39.2%), or reduced ejection fraction (21.6%), and a high incidence of pulmonary dysfunction (60.8%) including pulmonary edema, respiratory distress, and cyanosis. Individuals received one or more antihypertensives (58.8%) and heart failure treatments (52.9%) to address their presenting symptoms, while some also received bisphosphonates (54.9%) to address calcifications (Figure 2 and Table S2).

Imaging modalities used for the detection of calcifications, detailed in Table 2G, included echocardiography, ultrasound, plain radiographs, and CT. Only 9 (17.6%) underwent CT angiography, which may explain the low incidence of arterial stenosis recorded in this cohort (15.7%).

Surprisingly, one-quarter (25.5%) of GACI-only individuals had skeletal findings, including 8 with a medical record of metaphyseal abnormalities and/or rickets. Seven of the 8 individuals had a history of bisphosphonate therapy, with 6 treated with etidronate and 1 with alendronate. While dates of first skeletal symptoms were missing in some cases, we can confirm that bisphosphonate therapy commenced prior to the development of rickets/metaphyseal complications in 4 individuals.

#### GACI and ARHR2

This cohort (*n* = 22; 26.2%) consisted of individuals diagnosed with GACI who survived beyond infancy and were diagnosed with ARHR2 by the time of last follow-up. Thus, this group gives us insight into the natural history and evolution of the disease. The age of onset and incidence of cardiovascular manifestations in this cohort were similar to those with a GACI-only diagnosis, but with comparatively reduced severity that was ultimately reflected in survival status. These patients were subsequently diagnosed with ARHR2 on the basis of biochemical, radiographic, and/or clinical signs of rickets. The majority of individuals in this cohort (59.1%) had siblings either living or deceased with a diagnosis of GACI.

This cohort primarily presented prenatally or in infancy with cardiovascular complications. The median age at symptom onset was 7 d and median age at diagnosis was 30.5 d. There was a similar incidence of abnormal prenatal ultrasound findings (59.1%) compared with the GACI-only cohort (60.8%), primarily calcification of arteries (40.9%) and effusions (27.3%). Eleven (50%) individuals in this group were born prematurely at less than 37 wk’ gestation. Postnatally, they manifested arterial (100%) and aortic (86.4%) calcification with diverse cardiovascular complications (72.7%), including hypertension (45.5%) and heart valve defects (22.7%).

Though the incidence of cardiovascular disease (72.7%) was comparable with that of the GACI-only cohort (80.4%), the relatively lower need for medical interventions (Figure 2) and higher survival status (Table 1) in the GACI-ARHR2 cohort suggest milder disease severity. For example, at initial presentation, compared with the GACI-only cohort, there was a similar incidence of hospitalization (86.4% vs 88.2%), but a relatively lower requirement for mechanical ventilation (36.4% vs 80.4%). This was associated with a lower overall incidence of cardiac failure (9.1% vs 39.2%) and reduced ejection fraction (9.1% vs 21.6%), though a higher incidence of cardiac valve or myocardial calcification (50.0% vs 31.4%) was noted. A relatively higher use of bisphosphonates was observed in this cohort compared with those with GACI-only (68.2% vs 54.9%, respectively).

All who survived the critical period of infancy subsequently developed musculoskeletal complications and were diagnosed with ARHR2 at a median age of 3.4 yr (range 0.04-17; Table 1, Figure S5). Radiographic rickets was documented in 95.5% of individuals, with bowed extremities in 68.2% and metaphyseal irregularities in 59.1%. Related clinical symptoms included pain (68.2%), short stature (36.4%), and gait abnormality (31.8%). Most required active vitamin D treatment (81.8%) and phosphate supplements (68.2%) for management of rickets. Periarticular calcification was also prevalent (59.1%) and likely contributed to joint pain (27.3%). Few individuals had fractures or vertebral fusion noted in their medical history (each *n* = 2), which may be attributable to a younger ARHR2 population or a lack of imaging.^11^ In contrast to the GACI-only cohort, the most prevalent other organ involvements were related to hearing (68.2%, 13 with conductive hearing loss) and the gastrointestinal tract (63.6%).

Notably, 1 individual with a diagnosis of GACI and ARHR2 died of cardiorespiratory arrest around the age of 5 yr. At birth, this infant presented with pulmonary hypertension and cyanosis, with imaging evidence of ectopic calcification of the aortic arch, ascending aorta, and coronary, pulmonary, and subclavian arteries. With the exception of the subclavian artery, vascular calcifications reportedly had resolved by 2.5 yr of age. Medical history was also notable for left ventricular hypoplasia and absent mitral valve. The patient had developed rickets with documented gait abnormality, pain, and fracture, and was on calcitriol treatment at the time of death.

#### ARHR2-only

Given the high incidence of clinically significant arterial disease, it is expected that individuals with ENPP1 Deficiency would present with symptoms/signs around birth. However, there are published cases of individuals who first presented with musculoskeletal complications and were ultimately diagnosed with ARHR2 in childhood or adulthood.^12^^,^^13^ Eleven such “ARHR2-only” individuals were identified in our analysis. A key question about these individuals is whether they had subclinical evidence of GACI.

The 11 individuals in this study with ARHR2-only were diagnosed across a broad age range, with notable diagnostic delay (median age at symptom onset was 3.6 yr [range 1.5-4.9 yr]; median age at diagnosis 11.5 yr [range 4.5-24.7 yr]).

Unsurprisingly, the medical burden in this cohort was largely related to musculoskeletal complications. All individuals had rickets (100%), along with bowed extremities (72.7%) and history of pain (54.5%) or joint pain (27.3%). Three individuals also had a history of periarticular calcification. Overall, there was a high utilization of active vitamin D and phosphate supplements (both 63.6%). In this cohort, among those alive at last follow-up, the median age at last follow-up was 18.0 yr (range 3.3-58.3 yr) and none were hospitalized over the follow-up period.

**Figure 3:**
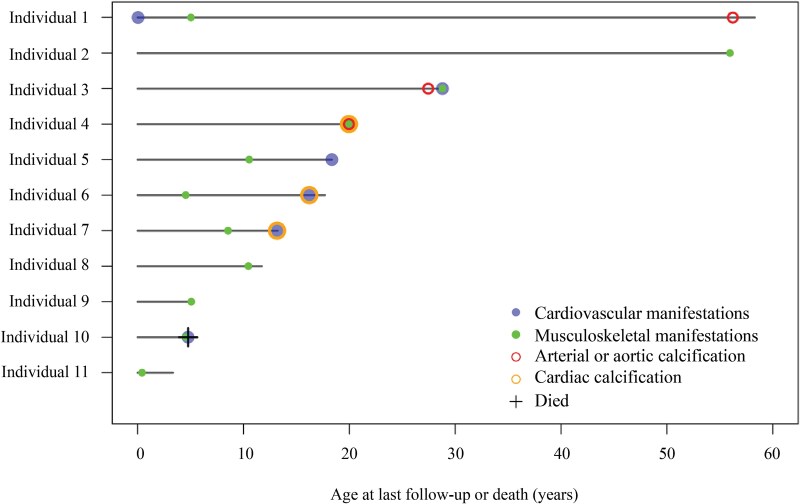
Timing of manifestations (presentation, age at last follow-up or death) in individuals with an ARHR2-only diagnosis. Where no date of event was provided, the age at last follow-up was used to mark the presence of the manifestation. Abbreviation: ARHR2, autosomal recessive hypophosphatemic rickets type 2.

Extra-skeletal manifestations, including cardiovascular signs and symptoms, were evident across individuals’ medical histories. Among those who underwent ultrasound or CT scans, arterial (*n* = 3) or cardiac valve calcification (*n* = 3) were identified in the absence of a GACI diagnosis. Figure 3 shows the age at which cardiovascular manifestations were first identified in the ARHR2-only population. Seven individuals (63.6%) had cardiovascular manifestations, most commonly hypertension (*n* = 4) and heart valve involvement (*n* = 4). In individuals with ages of event recorded, 1 had cardiac calcification and cardiovascular manifestations at age 16 yr, another had cardiovascular manifestations in infancy with vascular calcifications documented at age 56 yr, another had vascular calcification at age 27 yr, and 1 had cardiac calcification at age 13 yr. Relatively fewer individuals had echocardiograms (36.4%) compared with the GACI and ARHR2 individuals (77.3%; Table 2G).

Most (81.8%) had other organ manifestations: 45.5% with gastrointestinal symptoms, 36.4% with hearing complications, and 27.3% with renal complications, including nephrocalcinosis in 1 individual, which is a known side effect of calcitriol and phosphate treatments.^14^

An individual with ARHR2-only in the study died at age 4 yr, 9 mo with bronchopneumonia and multifocal myocardial fibrosis. Their medical history included bowed extremities and metaphyseal cupping noted around 4.5 yr. Approximately 2 mo later, heart failure, cardiac arrhythmia, cyanosis, and seizure were noted. A hippocampal malformation known as “granule cell dispersion” was found on autopsy; this finding has been associated with temporal lobe epilepsy but has also been described as a nonspecific normal variant.^15^

## Discussion

This analysis sheds light on the incidence, burden, and evolution of clinical manifestations of ENPP1 Deficiency due to variants in *ENPP1* from the largest retrospective chart review to date. The recorded medical complications in affected individuals over time highlight the diagnostic challenges and clinical course of the condition, underscoring the limitations of the diagnostic terms GACI and ARHR2 in capturing the full spectrum of this multisystem genetic disease.

In this population of 84 individuals with ENPP1 Deficiency, the vast majority (76%) presented in infancy with cardiac insufficiency and respiratory distress requiring acute inpatient care. A cumulative incidence analysis demonstrated that the onset of cardiovascular complications corresponded to the onset of vascular calcification. Hypertension and heart failure were the most prevalent cardiovascular symptoms recorded at any time point in individuals with GACI, while the most prevalent signs on imaging were arterial and aortic calcification, left ventricular hypertrophy, pericardial effusion, and cardiomegaly. In those with GACI, periarticular and other organ calcification may be an incidental finding, as the primary burden of disease appeared to be cardiovascular in nature.

Importantly, we demonstrate that the majority of individuals with GACI had evidence of disease observable on prenatal ultrasound. Polyhydramnios and nonimmune hydrops fetalis may be linked to vascular dysfunction caused by ENPP1 Deficiency through potential increased central venous pressure, changes in oncotic pressure, and neurohormonal changes that may result in amniotic fluid accumulation.^9^^,^^16^ Prenatal onset may not be predictive of mortality, as a similar incidence of fetal ultrasound anomalies was reported in the GACI-only and GACI and ARHR2 cohorts.

Over 40% of individuals in this study died in infancy. In those with GACI who survived, 53% developed hypophosphatemic rickets during the follow-up period and were diagnosed with ARHR2 throughout childhood. In addition, 11 were diagnosed with ARHR2-only after infancy and without a prior history of GACI. The substantial delay between symptom onset and ARHR2 diagnosis in this cohort is not unexpected, as the manifestations of ARHR2 mimic other forms of hypophosphatemic rickets, osteomalacia, and osteoarthritis.^17^ Taken together, we estimated approximately 92% of GACI survivors will develop musculoskeletal complications by 20 yr of age. Of note, the role of bisphosphonate therapy precipitating skeletal complications cannot be ruled out in some; in the GACI-only cohort, 88% of those with metaphyseal abnormalities/rickets had received bisphosphonates. This suggests, therefore, that early skeletal findings in this GACI-only cohort are likely iatrogenic due to bisphosphonate therapy. Skeletal abnormalities seen with prolonged etidronate therapy include rickets and osteomalacia through a non-FGF23-mediated mechanism.^18^

We confirm the most prevalent signs and symptoms of ARHR2 were related to hypophosphatemic rickets and osteomalacia, including bowed legs, metaphyseal abnormalities, pain, short stature, abnormal gait, and fracture. We demonstrate that the burden of ARHR2 extends beyond the bone to include periarticular calcification, as well as enthesopathy and vertebral fusion, typically found in an older population.

A key finding was that historical or current cardiovascular manifestations were prevalent among the ARHR2 population, with or without a history of GACI, and 2 individuals diagnosed with ARHR2 died during childhood. A cumulative incidence analysis showed that cardiovascular calcification, especially of the heart valves, continued to be identified in survivors beyond infancy. In individuals with an ARHR2-only diagnosis, 63.6% had a medical history of cardiovascular complications. Few of these underwent echocardiography, but in those who did, nearly all had evidence of calcification. None from this subcohort underwent CT/MR angiography, so the incidence of arterial stenosis is likely underreported, highlighting an inconsistency in screening and management. Whereas the development of hypophosphatemia in surviving patients may promote the regression of arterial calcification, it likely does not ameliorate vascular stenosis.^6^^,^^19^ Thus regular monitoring of both calcifications and stenoses should be considered.

Beyond the cardiovascular and musculoskeletal systems, individuals across phenotypic diagnoses had significant other organ complications. A diagnosis of GACI was associated with a high incidence of pulmonary complications, likely a byproduct of poor left ventricular function causing pulmonary vascular congestion from reduced antegrade flow.^20^ A diagnosis of ARHR2 was associated with a high incidence of hearing loss, which has been attributed to abnormal remodeling of the middle ear ossicles.^21^^,^^22^ In addition, gastrointestinal, renal, and neurologic defects were documented across phenotypes. Taken together, these results paint a picture of the clinical evolution of ENPP1 Deficiency from severe, often fatal cardiovascular complications during infancy, to a primarily musculoskeletal phenotype in survivors, and with heterogenous multisystem complications across the lifetime.

While this analysis provides important information on the natural history of ENPP1 Deficiency, it has its limitations. The retrospective nature of this medical record analysis limits data consistency, quality, and completeness. Specifically, there were inconsistencies in the level of detail captured for dates of event (eg, birth or manifestation) and results (eg, specific biochemical results were not captured for all individuals with a diagnosis of ARHR2, nor prenatal findings for those with ARHR2 only in the CRFs). There are also irregularities in the way the diagnoses were attributed, with some patients with GACI noted to have rickets, but not formally diagnosed with ARHR2 (and similarly those with ARHR2 and cardiovascular manifestations but not diagnosed with GACI).

Lack of standardization across assessments (eg, imaging) is another challenge with this population. Absence of a report of a manifestation could not always be interpreted as an absence of the manifestation, as there were inconsistent assessments and levels of detail in documentation over the follow-up.

Furthermore, there was a relatively short duration of follow-up. Therefore, complications that take time to become clinically apparent or traditionally present during adulthood, such as enthesopathy, osteoarthritis, and spinal fusion, may be underrepresented.^23^ Other case reports in the literature have noted initial presentation of ARHR2 much later in life.^24–26^ Thus, it is likely that the true medical burden and milder spectrum of disease are not captured due to the limited follow-up.

## Conclusions

This study informs our understanding of GACI and ARHR2 as a phenotypic continuum of ENPP1 Deficiency with implications for diagnosis and lifelong management. The high mortality of the disease and multisystem burden, in addition to the diagnostic delay observed in this study, point to the need for improved ENPP1 Deficiency diagnostic awareness and multidisciplinary patient monitoring.

It is well established that fetal or infantile arterial calcification is the diagnostic hallmark of GACI, which results in nonspecific cardiovascular complications. As these complications may precede the detection of arterial calcification, it is important for fetal and neonatal medicine providers to be aware of GACI as a differential diagnosis for fetal effusions, hydrops fetalis, and polyhydramnios, and infantile heart failure or hypertension of undetermined etiology. Emphasis should be put on the importance of prenatal calcification findings, with the development of tools to help alert clinicians to a possible diagnosis of GACI.

As the phenotype of ENPP1 Deficiency evolves from GACI to ARHR2, a multidisciplinary team including geneticists, cardiologists, endocrinologists, and bone specialists is recommended to monitor cardiovascular involvement and the development of hypophosphatemic rickets, and to inform treatment decisions. Our analysis suggests that cardiovascular workup should be considered even in patients with late presenting skeletal disease with no documented history of GACI. Ultimately, each patient’s care plan should be tailored to their presenting phenotype. Nonetheless, these population-level data shed light on the core manifestations of ENPP1 Deficiency and its presentation over the lifetime, reflecting a single pathobiology underlying the phenotypic diagnoses of GACI and ARHR2. These patients require considerable medical management of their presenting symptoms, pointing to an unmet need for disease-modifying therapies that address the multisystem manifestations of ENPP1 Deficiency.

## Supplementary Material

Supplementary_Appendix_ziaf019

## Data Availability

The data underlying this article are available in the article and in its online supplementary material.
