# Supplementary material for: Phenotypic characterization of ENPP1 deficiency: generalized arterial calcification of infancy and autosomal recessive hypophosphatemic rickets type 2
Source: JBMR Plus. 2025 Jan 30;9(5):ziaf019. doi: 10.1093/jbmrpl/ziaf019 (PMC11961066; doi:10.1093/jbmrpl/ziaf019)
Supplement: Supplementary_Appendix_ziaf019 [file supplementary_appendix_ziaf019.docx]

**SUPPLEMENTARY APPENDIX**

**Table S1. Siblings with a diagnosis of GACI.**

| **Event, n (%)** | **Overall (N=84)** | **GACI-only (n=51)** | **GACI and ARHR2 (n=22)** | **ARHR2-only (n=11)** |
| --- | --- | --- | --- | --- |
| **Stillborn/pregnancy terminated**  Yes  No  Unknown | 7 (8.3%)  57 (67.9%)  20 (23.8%) | 6 (11.8%)  33 (64.7%)  12 (23.5%) | 1 (4.5%)  18 (81.8%)  3 (13.6%) | 0 (0.0%)  6 (54.5%)  5 (45.5%) |
| **Living sibling(s)**  Yes  No  Unknown | 15 (17.9%)  59 (70.2%)  10 (11.9%) | 9 (17.6%)  34 (66.7%)  8 (15.7%) | 5 (22.7%)  17 (77.3%)  0 (0.0%) | 1 (9.1%)  8 (72.7%)  2 (18.2%) |
| **Deceased sibling(s)**  Yes  No  Unknown | 19 (22.6%)  54 (64.3%)  11 (13.1%) | 10 (19.6%)  36 (70.6%)  5 (9.8%) | 8 (36.4%)  12 (54.5%)  2 (9.1%) | 1 (9.1%)  6 (54.5%)  4 (36.4%) |
| **Living sibling(s) in study**  Yes  No  Unknown | 21 (25.0%)  4 (4.8%)  59 (70.2%) | 11 (21.6%)  1 (2.0%)  39 (76.5%) | 9 (40.9%)  2 (9.1%)  11 (50.5%) | 1 (9.1%)  1 (9.1%)  9 (81.8%) |

**Table S2. Treatments received.**

| **Characteristic** | **Overall (N=84)** | **GACI-only (n=51)** | **GACI and ARHR2 (n=22)** | **ARHR2-only (n=11)** |
| --- | --- | --- | --- | --- |
| **Antihypertensive treatments** | | | | |
| Any | 41 (48.8%) | 30 (58.8%) | 8 (36.3%) | 3 (27.3%) |
| Angiotensin-converting enzyme (ACE) inhibitors | 22 (26.2%) | 16 (31.4%) | 5 (22.7%) | 1 (9.1%) |
| Beta blockers | 21 (25.0%) | 15 (29.4%) | 4 (18.2%) | 2 (18.2%) |
| Calcium channel blockers | 21 (25.0%) | 17 (33.3%) | 4 (18.2%) | 0 (0.0%) |
| Thiazide diuretics | 16 (19.0%) | 10 (19.6%) | 5 (22.7%) | 1 (9.1%) |
| Vasodilators | 9 (10.7%) | 7 (13.7%) | 2 (9.1%) | 0 (0.0%) |
| Adrenergic receptor blockers | 6 (7.1%) | 4 (7.8%) | 2 (9.1%) | 0 (0.0%) |
| Angiotensin receptor blockers | 4 (4.8%) | 2 (3.9%) | 1 (4.5%) | 1 (9.1%) |
| Renin inhibitors | 1 (1.2%) | 0 (0.0%) | 0 (0.0%) | 1 (9.1%) |
| Mineralocorticoid receptor antagonist | 1 (1.2%) | 1 (2.0%) | 0 (0.0%) | 0 (0.0%) |
| **Mechanical ventilation** | | | | |
| Any | 48 (57.1%) | 41 (80.4%) | 8 (36.4%) | 0 (0%) |
| **Heart failure treatments** | | | | |
| Any | 38 (45.2%) | 27 (52.9%) | 10 (45.5%) | 1 (9.1%) |
| Diuretics | 18 (21.4%) | 10 (19.6%) | 7 (31.8%) | 1 (9.1%) |
| ACE inhibitors | 14 (16.7%) | 9 (17.6%) | 5 (22.7%) | 0 (0.0%) |
| Beta blockers | 13 (15.5%) | 10 (19.6%) | 3 (13.6%) | 0 (0.0%) |
| Aspirin | 12 (14.3%) | 7 (13.7%) | 5 (22.7%) | 0 (0.0%) |
| Calcium channel blockers | 6 (7.1%) | 3 (5.9%) | 3 (13.6%) | 0 (0.0%) |
| Digoxin | 3 (3.6%) | 3 (5.9%) | 0 (0.0%) | 0 (0.0%) |
| Hydralazine | 2 (2.4%) | 2 (3.9%) | 0 (0.0%) | 0 (0.0%) |
| Mineralocorticoid receptor antagonist | 1 (1.2%) | 1 (2.0%) | 0 (0.0%) | 0 (0.0%) |
| Nitrate | 1 (1.2%) | 1 (2.0%) | 0 (0.0%) | 0 (0.0%) |
| **Bisphosphonate treatments** | | | | |
| Any | 45 (53.6%) | 28 (54.9%) | 15 (68.2%) | 2 (18.2%) |
| Etidronate | 27 (32.1%) | 14 (27.5%) | 11 (50.0%) | 2 (18.2%) |
| Pamidronate | 22 (26.2%) | 16 (31.4%) | 6 (27.3%) | 0 (0.0%) |
| Risedronate | 8 (9.5%) | 5 (9.8%) | 3 (13.6%) | 0 (0.0%) |
| Alendronate | 2 (2.4%) | 2 (3.9%) | 0 (0.0%) | 0 (0.0%) |
| **Rickets treatments** | | | | |
| Vitamin D (active) | 36 (42.9%) | 11 (21.6%) | 18 (81.8%) | 7 (63.6%) |
| Phosphate | 25 (29.8%) | 3 (5.9%) | 15 (68.2%) | 7 (63.6%) |

**Figure S1. Cumulative incidence of ectopic calcification in the first year of life.**

**
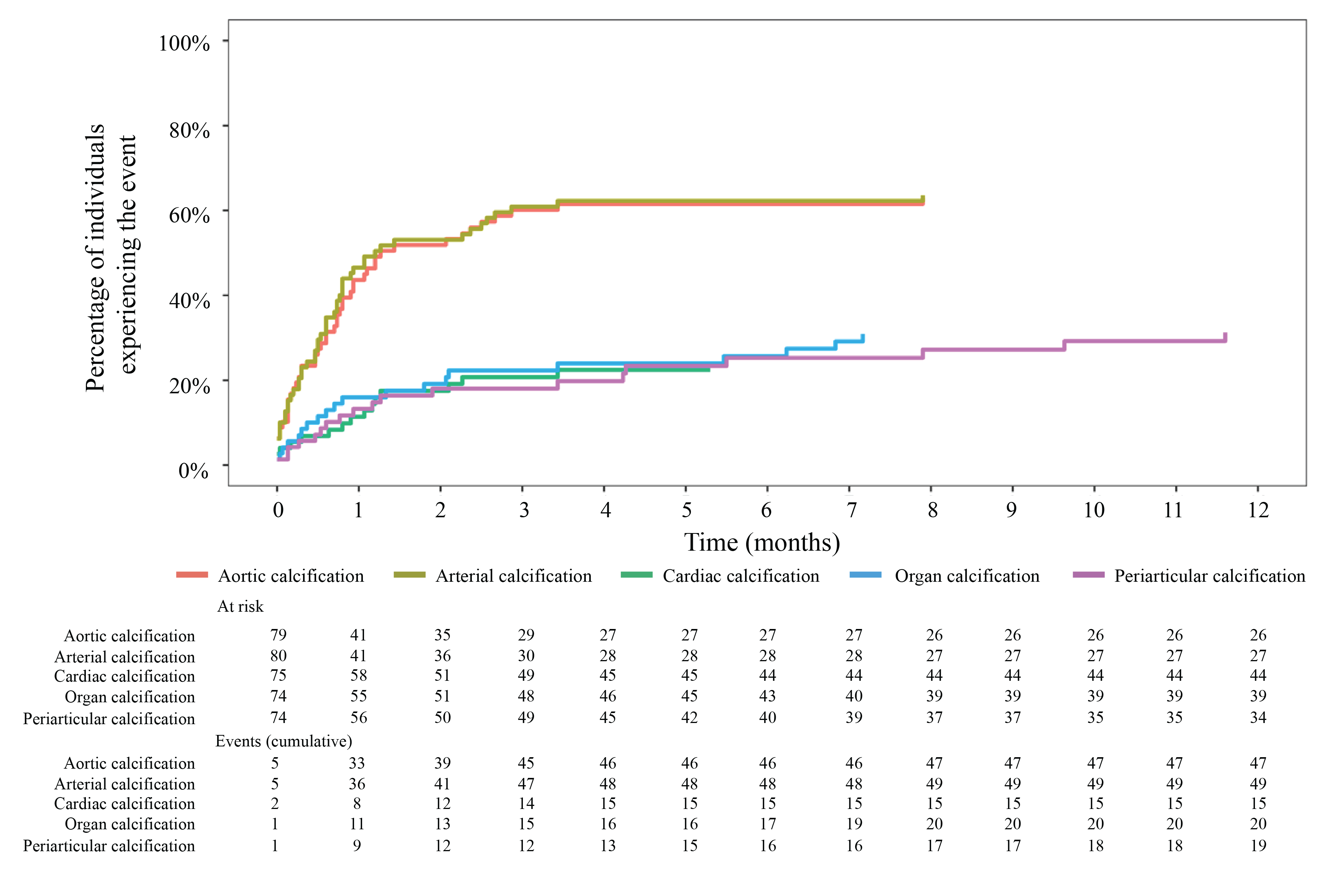
**

**Figure S2. Cumulative incidence of cardiovascular, musculoskeletal, and other organ manifestations in the first year of life.**

**
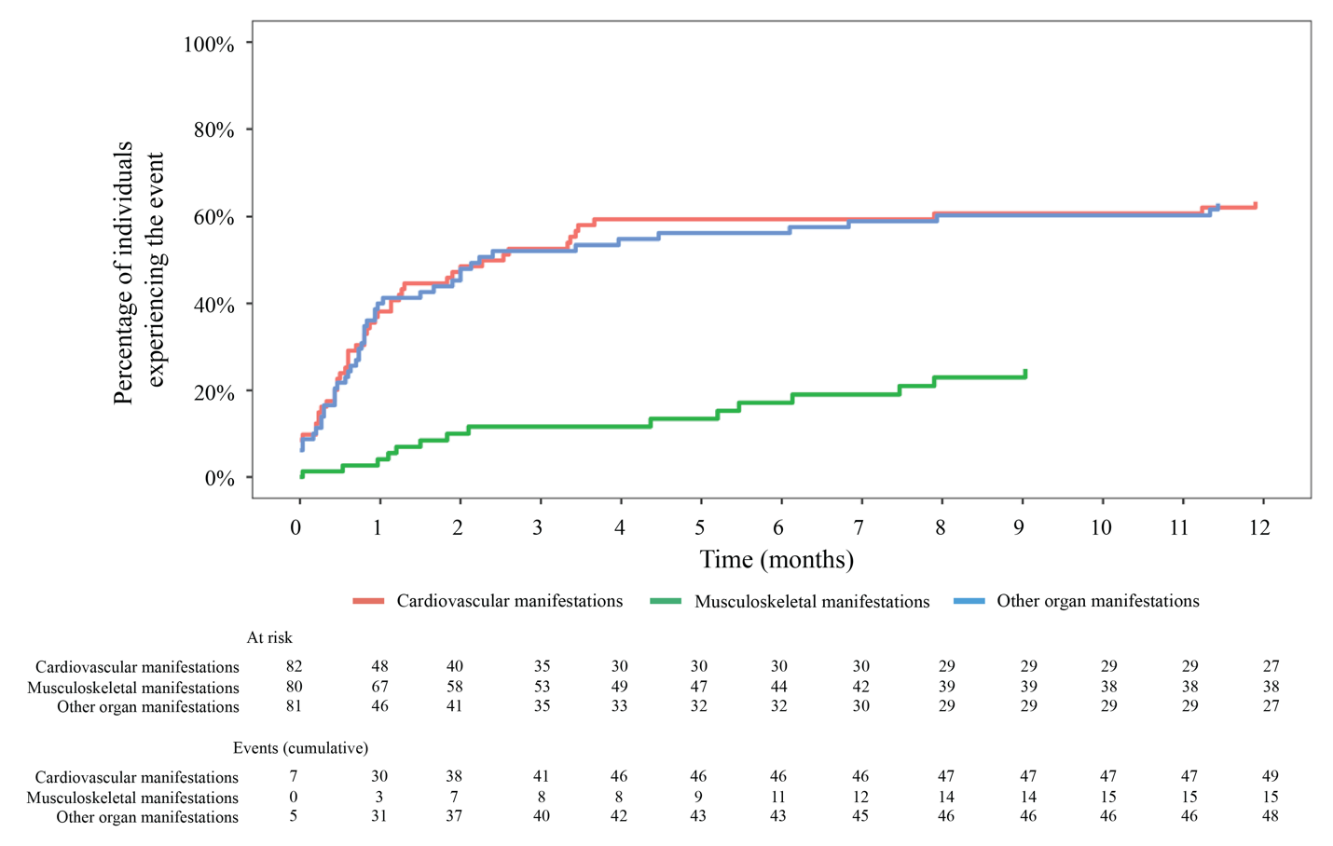
**

**Figure S3. Cumulative incidence of ectopic calcification over the first 55 years of life.**


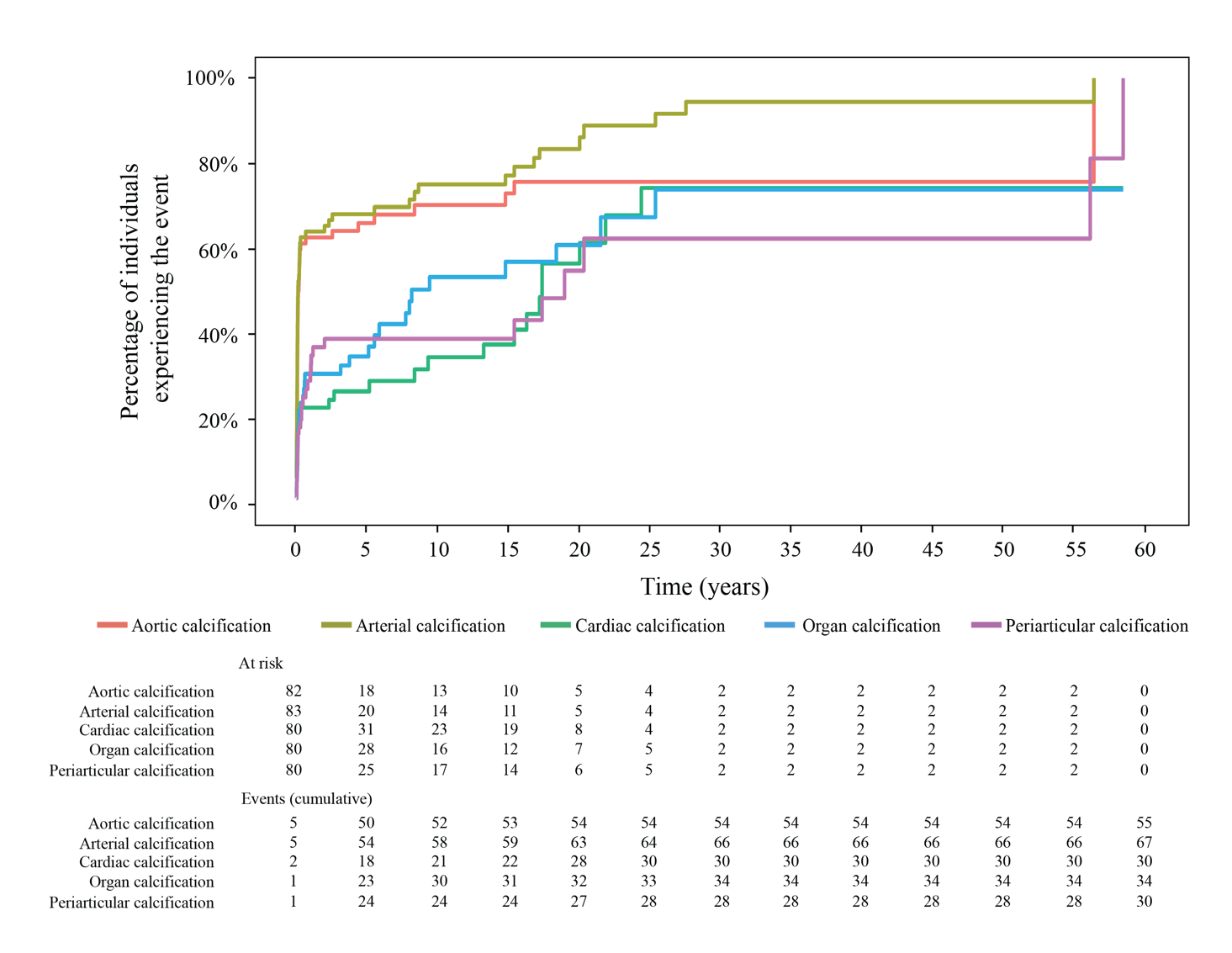


**Figure S4. Cumulative incidence of cardiovascular, musculoskeletal, and other organ manifestations over the first 55 years of life.**

**
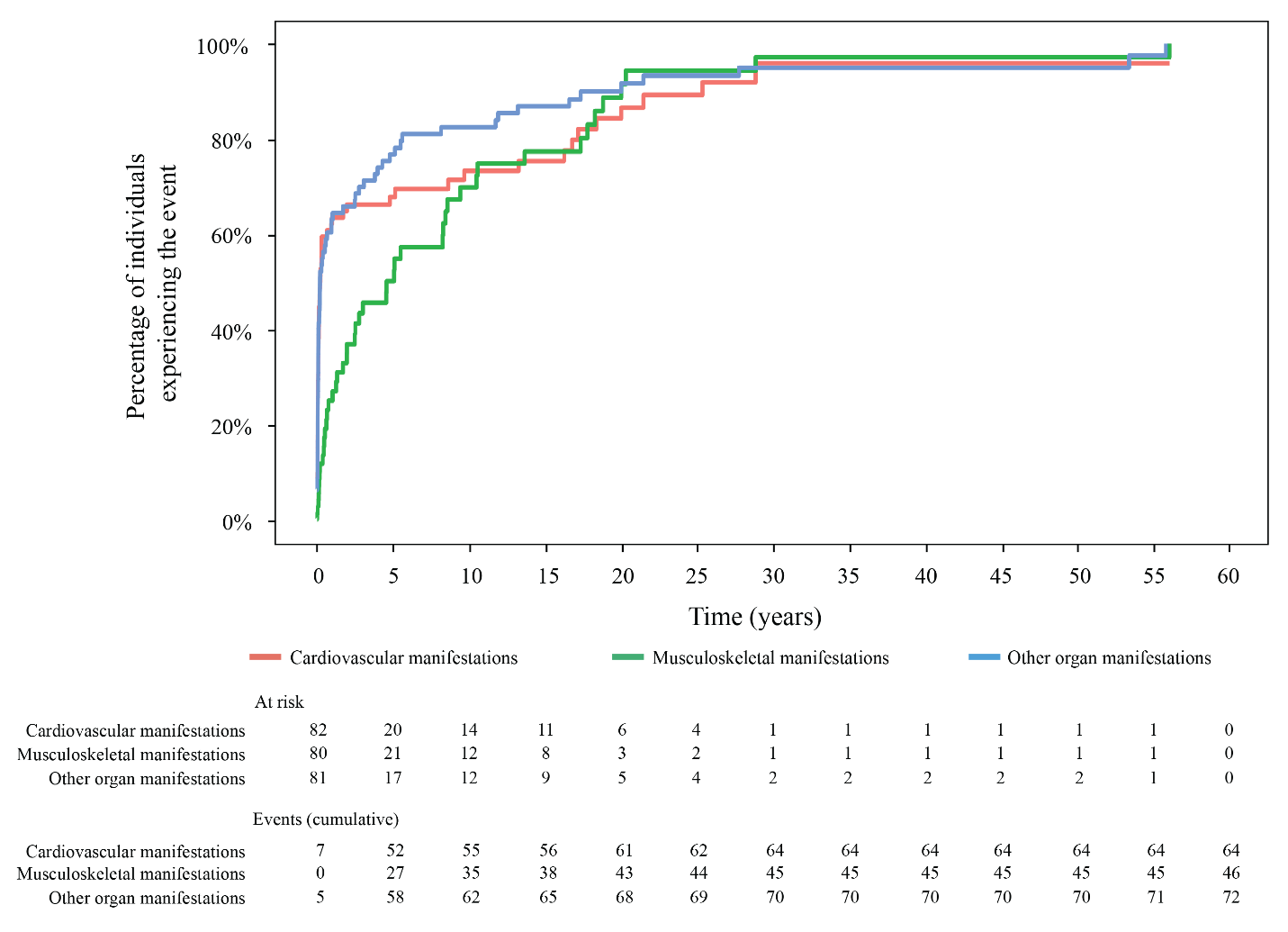
**

At 20 years, 87% (95% CI: 73%-93%) had cardiovascular manifestations, 92% (95% CI: 76%-97%) had musculoskeletal manifestations, and 92% (95% CI: 82%-96%) had other organ manifestations. At 55 years, 96% (95% CI: 78%-99%) had cardiovascular manifestations, 97% (95% CI: 81%-100%) had musculoskeletal manifestations, and 98% (95% CI: 87%-100%) had other organ manifestations.

**Figure S5.** **Age at diagnosis of GACI and ARHR2.**


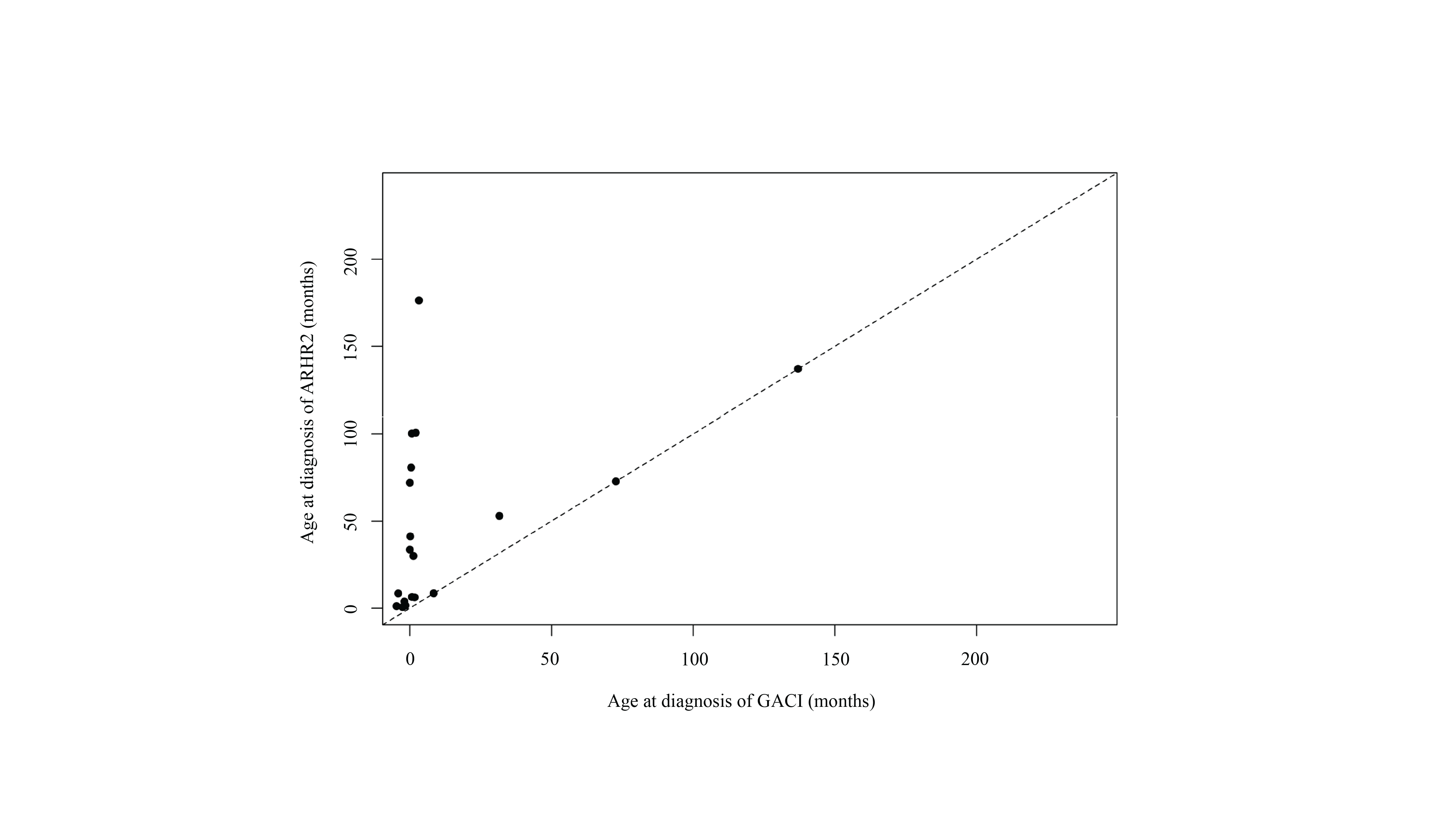


Median age of diagnosis of ARHR2 in individuals with GACI and ARHR2 was 41.2 months.
